# Genomic selection strategies for the German Merino sheep breeding programme – A simulation study

**DOI:** 10.1111/jbg.12897

**Published:** 2024-09-11

**Authors:** Rebecca Martin, Torsten Pook, Jörn Bennewitz, Markus Schmid

**Affiliations:** ^1^ Institute of Animal Science University of Hohenheim Stuttgart Germany; ^2^ Animal Breeding and Genomics Wageningen University and Research Wageningen the Netherlands

**Keywords:** breeding planning, breeding value estimation, genetic gain, genotyping

## Abstract

Genomic selection is widely implemented in livestock breeding programmes across species. Its potential is also evident for sheep breeding; however, it has several limitations, particularly because of the high genetic diversity across and within sheep breeds. In Germany, the predominant sheep breed is the Merino sheep. Until now, there has been no use of genomic selection in the German Merino sheep breeding programme. In this simulation study, different genomic selection strategies were compared with a reference scenario with a breeding value estimation based on pedigree BLUP. A simplified version of the German Merino sheep breeding programme, including a health and a production trait in the breeding goal, was simulated via the R package Modular Breeding Program Simulator (MoBPS). Real genotype data were used to create a population specific simulation. The reference scenario was compared with several alternative scenarios in which selection was based on single‐step GBLUP (ssGBLUP) breeding value estimation with varying genotyping strategies. In addition to scenarios in which all male and all male plus all female lambs were genotyped, scenarios with a preselection of lambs, that is only a certain proportion (top 25%, top 50%) genotyped, were simulated. The results revealed that genetic gain increased with increasing numbers of available genotypes. However, marginal gains decreased with increasing numbers of genotypes. Compared with the reference scenario, genotyping the top 25% of male lambs increased the genetic gain for the breeding ram population by 13% for both traits whereas genotyping the top 50% of male lambs or all male lambs led to increases of 18% (17%) or 26% (21%) for the health (production) trait, respectively. The potential of genotyping females in addition to male lambs was less evident on the male side with no significant differences between the scenarios with different proportions of genotyped females. The results have shown that genomic selection can be a valuable tool to increase genetic gain in the German Merino sheep population and that the genotyping of a certain proportion of animals might lead to substantial improvement over pedigree‐based breeding value estimation. Nevertheless, further studies, especially economic evaluations, are needed before practical implementation.

## 1 INTRODUCTION

In Germany, the total sheep population is approximately 1.5 million, 70% of which are Merino sheep. Income is generated mainly by governmental subsidies for landscape management and conservation grazing. Lamb meat sales account for only approximately 40%, whereas wool generates <2% of the total income (Mendel, 2022). Sheep breeding in Germany is divided into herdbook herds (breeding nucleus), in which genetic gain is generated, and production herds. Herdbook sheep account for only a minor proportion of the total sheep population. For more details about the German sheep farming system and the Merino sheep breeding programme, see Martin et al. (2023). Since 2014, there has been a pedigree‐based BLUP breeding value estimation for the Merino sheep breed. However, selection is still often based on phenotypes and breeders tend to rely on their preferences mainly regarding the sheep's body conformation and tend to preselect lambs before the official selection steps of licensing on the male side and herdbook registration on the female side. Additionally, until now, artificial insemination has not been used, and ram rotation between farms has not been practiced very often. This might maintain high genetic diversity due to a low selection intensity and a small number of offspring per individual (Schmid et al., 2023). However, such breeding structures limit genetic gain.

Genomic selection (GS) has not yet been applied to German sheep breeding. Several studies have also proven the potential of GS for sheep breeding, for example in Astruc et al. (2022) and Duchemin et al. (2012) for the French Lacaune dairy sheep breed. The success of a GS breeding programme relies to a large extent on the size and quality of the reference population, as reviewed by Rupp et al. (2016). For the design of a reference population in sheep, significantly more animals need to be included in the reference set than for other livestock species due to the high genetic diversity, both between and within breeds (van der Werf et al., 2014). High genetic diversity was also observed by Schmid et al. (2023) in German Merino sheep. Setting up an adequate reference population proves to be difficult, especially in small populations with overall decreasing sheep numbers as well as insufficiently available phenotypes (Rupp et al., 2016). In such systems, single‐step GBLUP (ssGBLUP) (Legarra et al., 2009; Misztal et al., 2009) is an appropriate genetic evaluation method because it combines phenotypic, genotypic and pedigree‐based information without the need to genotype all animals (Rupp et al., 2016).

The aim of this simulation study was to investigate the potential of GS for German Merino sheep breeding. A simplified version of the German Merino sheep breeding programme was simulated, and different selection strategies or breeding value estimation methods (pedigree‐based, genomically optimized with varying shares of genotyping) were compared in terms of their effects on genetic gain, accuracy of breeding value estimation and inbreeding.

## 2 MATERIALS AND METHODS

### 2.1 Population simulation

**FIGURE 1:**
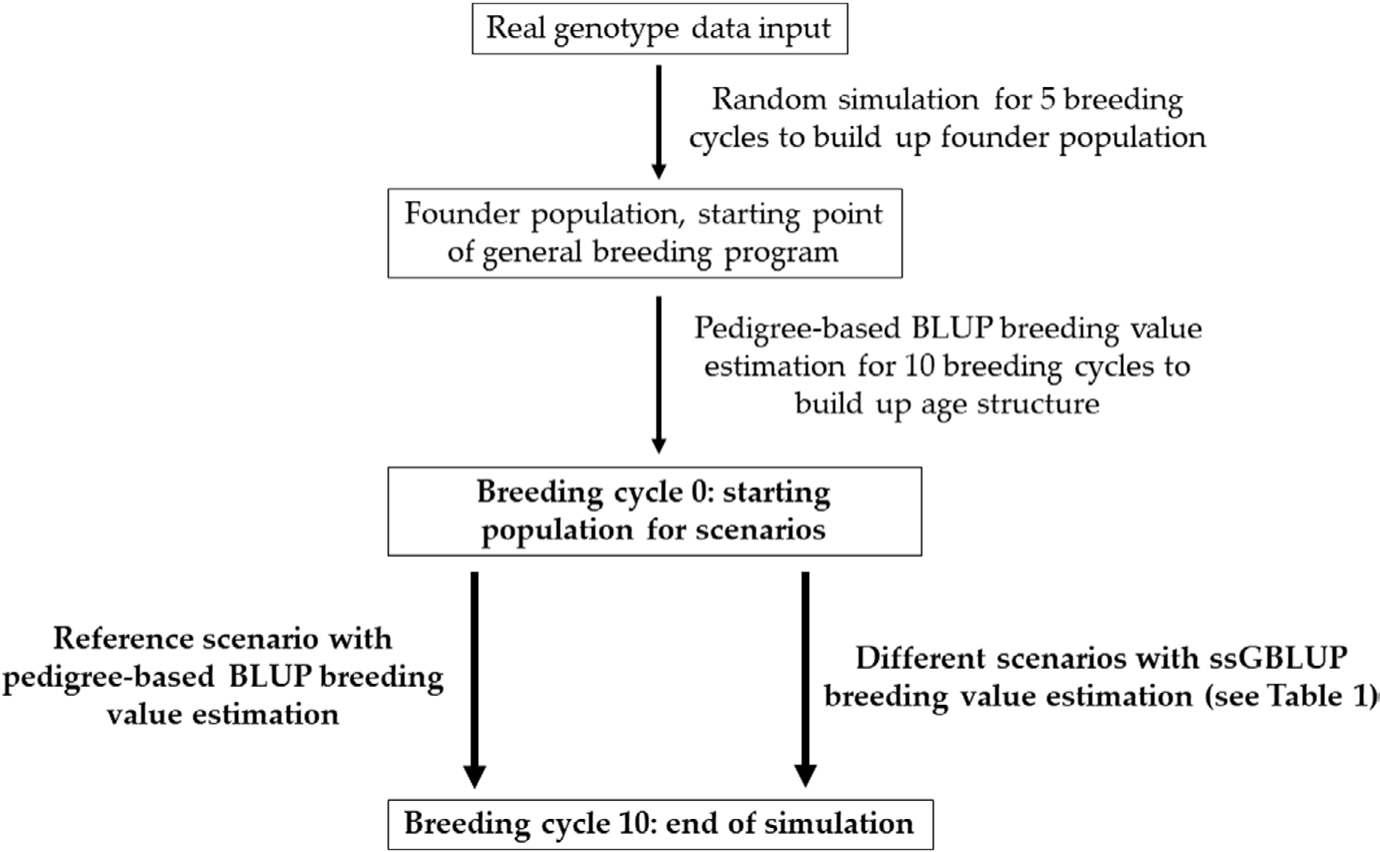
Schematic overview of the simulation timeline. The bold parts mark the simulation of breeding cycles 0–10, which are included in the evaluation.

The R package Modular Breeding Program Simulator (MoBPS) (Pook et al., 2020) was used to generate a simulated blueprint of the German Merino herdbook population (breeding nucleus) and to simulate the current breeding programme in a simplified way. Economic aspects were not considered. The R simulation script can be found at https://github.com/tpook92/MoBPS and in Data S1. The basic breeding programme setup (population‐specific parameters such as the number of lambs born, lamb sex ratio, litter size probabilities, etc.) was adopted from an earlier study (Martin et al., 2023). A detailed description of the simulation timeline is given in Figure 1. A realistic linkage disequilibrium (LD) structure of the starting population was obtained by the use of real genotype data from 785 German Merino sheep genotyped with a 50 K SNP chip (Illumina). To generate initial relatedness and a pedigree structure, individuals were randomly mated for five generations. The resulting founder population included 469 breeding rams and 7185 breeding ewes. This corresponds to the number of registered herdbook Merino sheep in Baden‐Wuerttemberg and Bavaria from January 2022. Animals registered in these two federal states account for the majority of the German herdbook Merino sheep and thus constitute a representative sample. The input genotype data were checked for population stratification in the founder population, but no clusters were detected in the PCA plot (data not shown). Thus, no further precorrection in the model for breeding value estimation was applied. After the founder population was simulated, the breeding programme was started. Ten breeding cycles with selection on the basis of a pedigree BLUP breeding value estimation were simulated as burn‐in to establish a realistic population structure. To obtain a realistic age structure, animals were culled randomly with a given probability depending on their age, analogous to the methodology of Büttgen et al. (2020), which is based on the data of all rams licensed in Bavaria between 2000 and 2021, see Martin et al. (2023) for details. Following these 10 breeding cycles, the starting point of the different scenarios described below was marked as breeding cycle 0. After that, the respective scenarios were simulated for another 10 breeding cycles (Figure 1).

### 2.2 Trait simulation

Two traits were simulated with 1000 purely additive underlying quantitative trait loci (QTLs) respectively. The QTLs were randomly selected from the SNP data. The traits were standardized to a population mean of 100 and a genetic variance of 10. The traits were intended to represent a health trait and a production trait and were simulated with heritabilities of 0.1 and 0.3 respectively. The genetic correlation between the traits was assumed to be −0.1, indicating that high‐yield individuals may be more susceptible to disease. The genetic parameters were adopted from the literature (Gauly et al., 2002; Medrado et al., 2021; Safari et al., 2005). The residual effects between traits were assumed to be uncorrelated. A detailed description of the QTL simulation protocol implemented in the MoBPS software and the resulting genetic correlation can be found in Chapter 15 of the MoBPS User Manual (Pook et al., 2023).

### 2.3 The reference breeding programme

**FIGURE 2:**
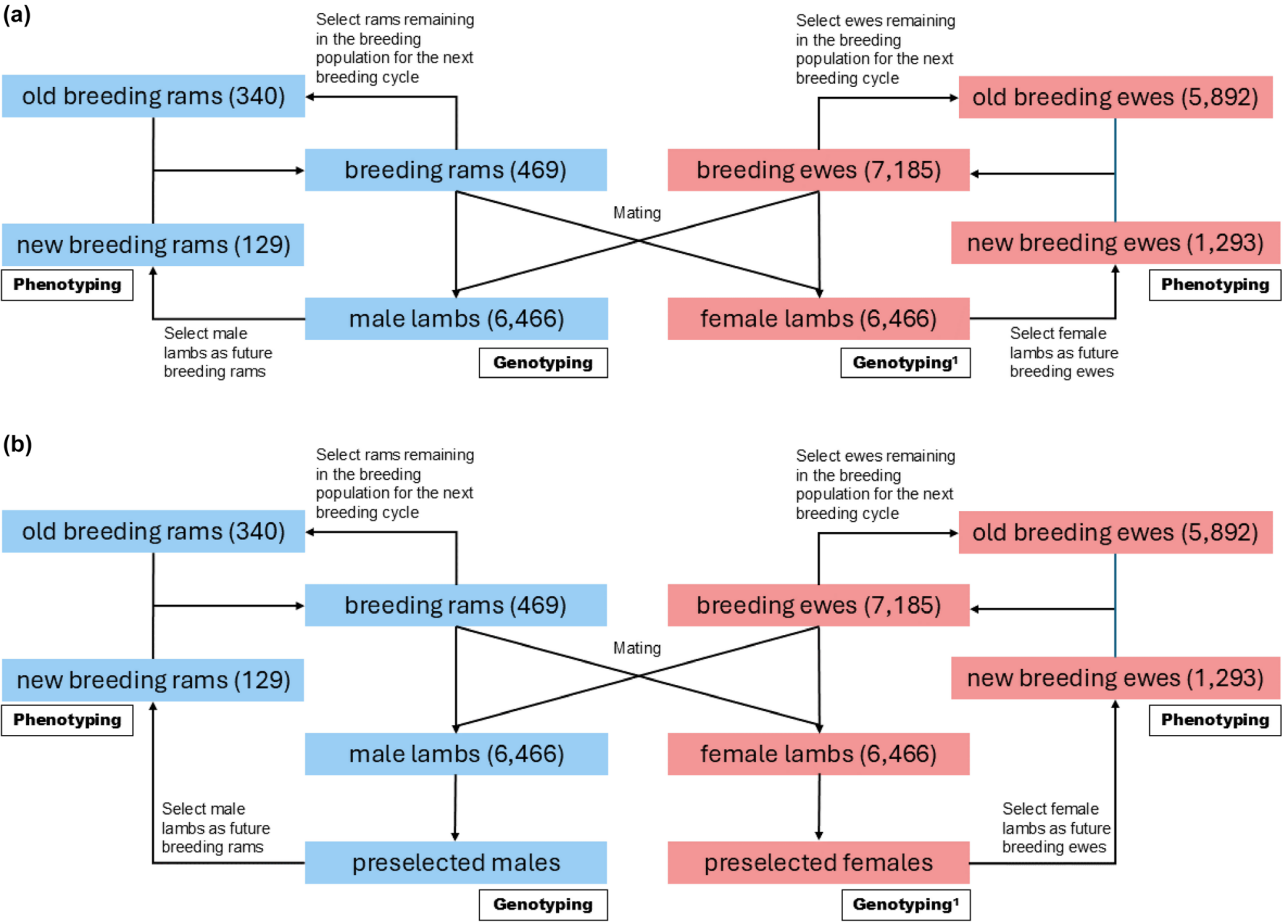
Schematic overview of the simulated sheep breeding population. The numbers in parentheses indicate the cohort sizes. The selection strategies differed between the simulated scenarios. Scenarios in which all male (and female, ^1^depending on the scenario) lambs were genotyped followed the scheme presented in panel (a). All other scenarios followed scheme (b), which included a preselection step to select a certain proportion of males (and females, ^1^depending on the scenario) to be genotyped. The pedigree‐based reference scenario was simulated analogously to panel (a); however, no individual was genotyped. For a detailed description of the scenarios, see Table 1. [Colour figure can be viewed at wileyonlinelibrary.com]

Figure 2 provides an overview of the simulated cohorts, selection steps and numbers of animals per cohort. An average number of 1.8 lambs born per ewe and lamb and a lamb loss rate of 0.1 before the selection stage were presumed. Because only the herdbook population was simulated, all male and female lambs produced (cohorts of male lambs and female lambs) were eligible as selection candidates. The animals that left the breeding programme were replaced with selected males and females. The cohort of active breeding rams was formed by combining the breeding rams of the previous breeding cycles that remain in the breeding population for the next breeding cycle, which were selected on the basis of their estimated breeding values (EBV) (old breeding rams) and the new breeding rams selected at a young age (new breeding rams). Analogously, the breeding ewe cohort consisted of breeding ewes from the previous breeding cycles that remain in the breeding population for the next breeding cycle and were selected on the basis of their EBVs (old breeding ewes) and new breeding ewes selected at a young age (new breeding ewes). The remaining cohorts and their role in the breeding programme were scenario‐specific and hence are described below for the respective scenarios.

The reference scenario (Ped) uses no genotype information which is in line with the current state of German Merino sheep breeding. The selection of individuals was based on a pedigree‐based BLUP breeding value estimation throughout the simulation in all selection steps. Individuals were selected based on a total merit index (TMI) with equal weights for both considered traits. For each trait, a BLUP animal model was applied to individuals of the last 10 breeding cycles. For each of these individuals, pedigree information was dated back to seven breeding cycles. The software mixBLUP (Vandenplas et al., 2022) was used to estimate breeding values in multi‐trait models. The trait means were modelled as fixed effects and the animal effect was modelled as a random effect, with the covariance structure modelled by the pedigree‐based relationship matrix. After the pedigree‐based breeding value estimation, the 2% best male lambs were selected from the cohort of male lambs as future breeding rams (cohort new breeding rams). On the female side, the best 20% of female lambs were selected from the cohort of female lambs as future breeding ewes (cohort new breeding ewes) (see Figure 2a). Phenotyping was conducted in the new breeding rams or new breeding ewe cohort. The phenotypes were considered as own performances of the individuals to indicate measurable traits later‐in life in a simple set‐up. No differences between the sexes at phenotyping were assumed. The selection of candidates already took place before phenotyping, since in the existing breeding programme, the most important selection step takes place at this point (cohort male or female lambs to cohort new breeding rams or new breeding ewes). Thus, their own phenotypes were not yet available for the selection candidates at the time of selection and were, therefore, not applicable as information for breeding value estimation.

### 2.4 Alternative scenarios

In contrast to the reference breeding programme, single‐step GBLUP (ssGBLUP) (Legarra et al., 2009; Misztal et al., 2009) breeding value estimation was performed in all selection steps of the alternative scenarios. The models for breeding value estimation in the alternative scenarios were identical to those in the reference scenario except for the covariance structure modelled by the ssGBLUP framework (Legarra et al., 2009; Misztal et al., 2009). To create an initial reference population for ssGBLUP selection, all active breeding rams (*n* = 469) at the starting point of the scenarios in breeding cycle 0 were assumed to be genotyped, that is their simulated genomic information was used for breeding value estimation. No sliding base of the reference population was applied over time; hence, the size of the reference population increased in each of the subsequent breeding cycles. Only the individuals selected in the cohorts of new breeding rams or new breeding ewes were genotyped and phenotyped, as phenotyping was not conducted before this stage in the breeding programme. Thus, the reference population grew by the constant number of selected animals with genotypes and phenotypes of 129 males (cohort new breeding rams) in scenarios with male lamb genotyping and additionally by 1293 females (cohort new breeding ewes) in scenarios with additional female genotyping.

In the alternative scenarios, different genotyping strategies were simulated including different proportions of genotyped males or individuals of both sexes. An overview of the scenarios is given in Table 1. Scenarios in which selection of future breeding animals was conducted via two‐step selection were simulated (Figure 2b). First, a certain proportion of lambs to be genotyped (depending on the simulated scenario, the top 25% of lambs in scenario GSTop25, and the top 50% of lambs in scenario GSTop50) were selected from the total male lamb cohort according to their EBV, that is their ssGBLUP EBV; however, with limited accuracy as no own phenotypes or genomic information was available, thus relying on parental information. This preselected cohort (preselected males) was then genotyped for 50 K SNPs. By using this additional information source, a second ssGBLUP breeding value estimation was subsequently conducted, and the 2% best males were selected from the cohort of genotyped preselected males to become breeding rams for the next breeding cycle (cohort of new breeding rams). In scenario GS100, all male lambs were selected to be genotyped, implying that selection was analogous to the reference scenario without a preselection step (Figure 2a).

**TABLE 1:** Description of the simulated scenarios.

Scenario	Description
Ped	Selection based on pedigree BLUP
GSTop25	ssGBLUP 25% top male lambs genotyped (preselected)
GSTop50	ssGBLUP 50% top male lambs genotyped (preselected)
GS100	ssGBLUP 100% all male lambs genotyped
GS100 + Top25	ssGBLUP 100% of male lambs genotyped + 25% top female lambs genotyped (preselected)
GS100 + Top50	ssGBLUP 100% of male lambs genotyped + 50% top female lambs genotyped (preselected)
GS100 + 100	ssGBLUP 100% of male lambs genotyped + 100% female lambs genotyped

In the previously described scenarios, no genotyping was assumed for the female lambs. To infer the effects of genotyping female individuals in addition to all male selection candidates, scenarios GS100 + Top25 and GS100 + Top50 were designed. They were simulated analogously to the protocol described above; however, 100% male plus the top 25% (top 50%) female lambs were assumed to be genotyped in scenario GS100 + Top25 (GS100 + Top50) (Figure 2b). In the last scenario, namely, GS100 + 100, genotypic information of all lambs alive, irrespective of sex, was used, thus again implying no preselection step (Figure 2a).

### 2.5 Statistical analysis

For each scenario, 100 independent runs were simulated. As displayed in Figure 1, only breeding cycles 0–10 were included in the evaluation of the scenarios. The mean genetic gain per breeding cycle was calculated as the average of the true breeding values per cohort. At the starting point of the evaluation in breeding cycle 0, the average of the true breeding values was scaled to zero.

The correlation of the true breeding values and the EBVs was assessed to evaluate the accuracy of the breeding value estimation (EBV accuracy). Inbreeding and the average level of heterozygosity per breeding cycle were assessed on the basis of information from 20 independent runs per scenario. Inbreeding levels between scenarios were compared by calculating the average kinship of the individuals of the breeding ram cohort in each breeding cycle. The development of average heterozygosity within a cohort between scenarios was calculated for the breeding ram cohort in each breeding cycle. The described metrics were calculated for all the scenarios and compared with those of the reference scenario as well as among the alternative scenarios. To detect significant differences between simulated scenarios paired two‐sample Student's t tests were performed with *p* < 0.05 indicating significant differences.

## 3 RESULTS

### 3.1 Genetic gain

The genetic gains in the breeding ram cohort and the breeding ewe cohort are displayed in Table 2 as the mean true breeding values across all runs after 10 breeding cycles for both simulated traits. The genetic gain for each breeding cycle (breeding cycles 1–10) is provided in Tables S1–S4. The increase in the number of genotyped lambs (selection candidates) was accompanied by an increase in genetic gain. Within each scenario, genetic gain increased with increasing numbers of animals in the reference populations, that is with every breeding cycle. Additionally, differences between scenarios became increasingly evident with every breeding cycle. Comparisons between the scenarios revealed the following findings. In general, all the GS scenarios significantly increased the genetic gain for both cohorts compared with the reference scenario with pedigree‐based breeding value estimation. Consequently, genetic gain was highest in scenario GS100 + 100, in which all selection candidates, irrespective of their sex, were genotyped and ssGBLUP was applied to all animals. For the breeding ram cohort, the genetic gains for both traits in this scenario were not significantly different from those in the scenarios in which only a preselected proportion of female lambs were genotyped in addition to all male lambs (GS100 + Top25, GS100 + Top50). However, for the breeding ewe cohort, genotyping 50% or all female lambs led to a significantly greater genetic gain than genotyping only 25% of female lambs. The highest marginal gain by increasing the proportion of genotyped individuals was observed between scenarios Ped and GSTop25, with a relative increase in genetic gain of approximately 13% for both traits for the breeding ram cohort. Marginal gains between scenarios subsequently decreased with increasing numbers of genotypes. Comparing between traits, the absolute genetic gain was more pronounced for the higher heritable production trait. However, the relative increase in genetic gain with GS was greater for the lower heritable health trait. For the breeding ram cohort, the increase in genetic gain for the health (production) trait after ten breeding cycles was 13% (13%), 18% (17%) and 26% (21%) if the top 25%, the top 50% or all male lambs were genotyped, respectively. Scenarios in which male and female genotypic information was available further increased genetic gain and was between 33% and 34% or 30% and 31% greater than that of the reference scenario in scenarios GS100 + Top25, GS100 + Top50 and GS100 + 100 for the health or the production trait for the breeding ram cohort after 10 breeding cycles. The impact of genotyping male lambs on genetic gain on the female side (breeding ewe cohort) was generally less pronounced. For the breeding ewe cohort, the increase in genetic gain after 10 breeding cycles for the health or production trait ranged from 5% or 4% (GSTop25) to 13% or 9% (GS100). Additionally, genotyping female lambs led to increases in genetic gain for the breeding ewe cohort of 20% (GS100 + Top25), 22% (GS100 + Top50) and 23% (GS100 + 100) for the health trait and 17% (GS100 + Top25) and 20% (GS100 + Top50 and GS100 + 100) for the production trait after 10 breeding cycles.

**TABLE 2:** Mean true breeding values (in genetic standard deviations [gSD]) across the 100 simulated runs for the health trait and production trait after 10 simulated breeding cycles for the reference scenario Ped (pedigree‐based breeding value estimation) and the alternative scenarios with genomic selection strategies GSTop25, GSTop50, GS100, GS100 + Top25, GS100 + Top50 and GS100 + 100 for the breeding ram cohort and the breeding ewe cohort.

	Breeding ram cohort		Breeding ewe cohort		
Scenario	Health trait	Production trait	Health trait		Production trait
Ped	1.380^a^ (0.160)	1.931^a^ (0.120)	1.397^a^ (0.108)	1.928^a^ (0.094)	
GSTop25	1.554^b^ (0.141)	2.183^b^ (0.122)	1.463^b^ (0.096)	2.012^b^ (0.096)	
GSTop50	1.632^c^ (0.163)	2.256^c^ (0.108)	1.502^c^ (0.108)	2.044^c^ (0.094)	
GS100	1.734^d^ (0.149)	2.335^d^ (0.115)	1.576^d^ (0.105)	2.099^d^ (0.089)	
GS100 + Top25	1.832^e^ (0.142)	2.518^e^ (0.116)	1.674^e^ (0.101)	2.264^e^ (0.097)	
GS100 + Top50	1.839^e^ (0.131)	2.534^e^ (0.123)	1.707^f^ (0.089)	2.312^f^ (0.102)	
GS100 + 100	1.852^e^ (0.155)	2.536^e^ (0.120)	1.721^f^ (0.096)	2.323^f^ (0.098)	

*Note*: For a detailed description of the scenarios, see Table 1. Superscript letters indicate significant differences between scenarios (*p* < 0.05).

### 3.2 Accuracy of breeding value estimation

The accuracy of the breeding value estimation per scenario after breeding cycle 10 is presented in Table 3 for the male lamb and breeding ram cohort as well as the female lamb and breeding ewe cohort. The mean accuracy for each breeding cycle (breeding cycles 1–10) is provided in Tables S5 to S12. The EBV accuracy was generally greater for the breeding ram and ewe cohort than for the lamb cohorts for both considered traits. Accuracies increased with increasing numbers of genotyped lambs, with the highest accuracies in the scenario with all male and female lambs genotyped (GS100 + 100). In line with the results for genetic gain, the highest marginal gain caused by the inclusion of additional information for breeding value estimation was observed when genotypic information was initially added for both male cohorts and both traits (between scenario Ped and GSTop25). Therefore, EBV accuracy increased by 12% (10%) for the male lamb cohort and by 11% (8%) for the breeding ram cohort for the health trait (production trait). However, on the female side, the highest marginal gain was observed when initially including female genotypes in addition to male genotypes (between scenarios GS100 and GS100 + Top25) in both female cohorts and for both traits. Accordingly, EBV accuracy increased by 11% (11%) for the female lamb cohort and by 9% (8%) for the breeding ewe cohort for the health (production) trait. Comparing between traits, the accuracies of the higher heritable production trait were greater than those of the lower heritable health trait.

**TABLE 3:** Mean accuracies of estimated breeding values (EBVs) and standard deviations (SD) for the health trait and production trait after the 10 simulated breeding cycles for the reference scenario (Ped) and the alternative scenarios with genomic selection strategies GSTop25, GSTop50, GS100, GS100 + 25, GS100 + Top50, and GS100 + 100 for the male lamb cohort and breeding ram cohort and for the female lamb cohort and the breeding ewe cohort.

	Male lamb cohort		Breeding ram cohort		Female lamb cohort		Breeding ewe cohort	
Scenario	Health trait	Production trait	Health trait	Production trait	Health trait	Production trait	Health trait	Production trait
Ped	0.410^a^ (0.026)	0.534^a^ (0.020)	0.526^a^ (0.047)	0.685^a^ (0.032)	0.413^a^ (0.024)	0.533^a^ (0.020)	0.560^a^ (0.025)	0.713^a^ (0.012)
GSTop25	0.459^b^ (0.025)	0.586^b^ (0.016)	0.585^b^ (0.046)	0.738^b^ (0.024)	0.445^b^ (0.027)	0.561^b^ (0.017)	0.590^b^ (0.022)	0.738^b^ (0.012)
GSTop50	0.495^c^ (0.024)	0.627^c^ (0.016)	0.596^bc^ (0.045)	0.752^c^ (0.027)	0.456^c^ (0.026)	0.573^c^ (0.018)	0.610^c^ (0.024)	0.752^c^ (0.010)
GS100	0.540^d^ (0.022)	0.682^d^ (0.013)	0.601^c^ (0.043)	0.759^c^ (0.027)	0.464^d^ (0.023)	0.578^d^ (0.016)	0.623^d^ (0.022)	0.760^d^ (0.010)
GS100 + Top25	0.572^e^ (0.020)	0.721^e^ (0.012)	0.621^d^ (0.037)	0.784^d^ (0.023)	0.513^e^ (0.019)	0.642^e^ (0.013)	0.679^e^ (0.017)	0.823^e^ (0.007)
GS100 + Top50	0.571^e^ (0.020)	0.722^ef^ (0.012)	0.624^d^ (0.036)	0.784^d^ (0.022)	0.538^f^ (0.020)	0.675^f^ (0.012)	0.682^ef^ (0.017)	0.826^f^ (0.008)
GS100 + 100	0.575^e^ (0.021)	0.725^f^ (0.012)	0.623^d^ (0.042)	0.787^d^ (0.023)	0.574^g^ (0.019)	0.724^g^ (0.013)	0.685^f^ (0.017)	0.827^f^ (0.009)

*Note*: For a detailed description of the scenarios, see Table 1. Superscript letters indicate significant differences between scenarios (*p* < 0.05).

### 3.3 Inbreeding and average level of heterozygosity

**FIGURE 3:**
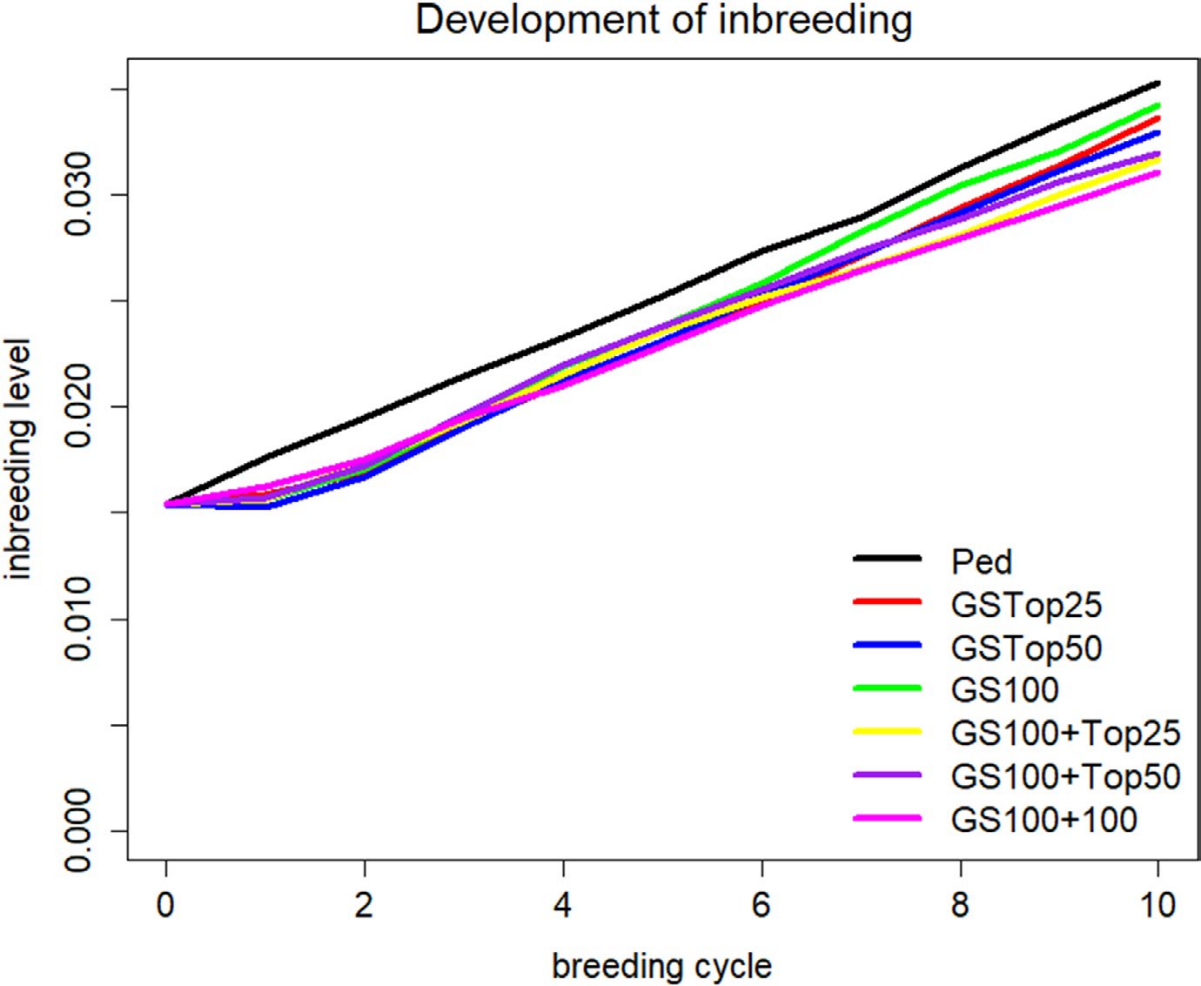
Level of inbreeding during the 10 considered breeding cycles for the breeding ram cohort for the reference scenario Ped (pedigree‐based breeding value estimation) and the alternative scenarios with genomic selection strategies GSTop25, GSTop50, GS100, GS100 + Top25, GS100 + Top50 and GS100 + 100. For a detailed description of the scenarios, see Table 1. [Colour figure can be viewed at wileyonlinelibrary.com]

**FIGURE 4:**
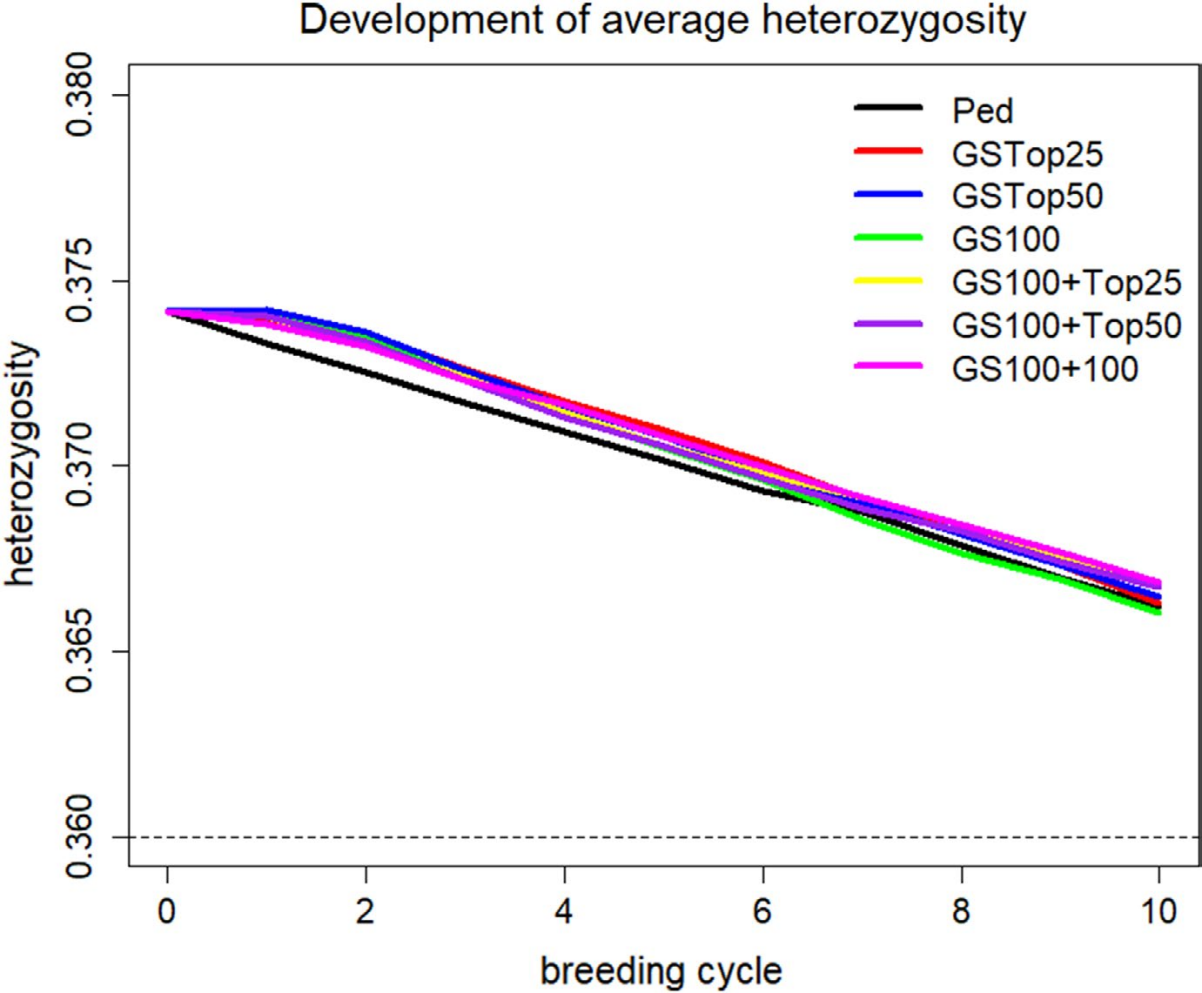
Development of the average level of heterozygosity during the 10 considered breeding cycles for the breeding ram cohort for the reference scenario Ped (pedigree‐based breeding value estimation) and the alternative scenarios with genomic selection strategies GSTop25, GSTop50, GS100, GS100 + Top25, GS100 + Top50 and GS100 + 100. For a detailed description of scenarios, see Table 1. [Colour figure can be viewed at wileyonlinelibrary.com]

Inbreeding within the breeding ram cohort in the respective scenarios is shown in Figure 3. Inbreeding was generally low and increased linearly in time, with greater inbreeding in the reference scenario than in the alternative scenarios. The development of the average level of heterozygosity during the simulation is shown in Figure 4 for the breeding ram cohort. In accordance with the results of the inbreeding rates, the average level of heterozygosity was greater for the GS scenarios than for the reference scenario on the basis of pedigree BLUP.

## 4 DISCUSSION

To date, GS has not been implemented in German Merino sheep breeding programmes, even though the potential of GS is evident for small ruminants and especially high for hard‐to‐measure traits (Rupp et al., 2016). To evaluate the potential of GS for the German Merino sheep breeding programme and to provide a basis for discussions on how to implement GS in this population, several scenarios incorporating different GS strategies (proportions of lambs genotyped) were simulated and compared with a reference scenario with pedigree‐based BLUP breeding value estimation.

### 4.1 Evaluation of the scenarios

Compared with the reference scenario, all the simulated scenarios with GS presented an increase in genetic gain in both the male and the female breeding populations. Genomic selection was more beneficial on the male side because of the higher replacement rate (20% in females vs. 2% in males) and the greater genetic contribution of males. This is due to the greater number of offspring from male than from female individuals. A greater benefit of GS for males than for females was also reported by Granleese et al. (2019). As expected for the GS scenarios, increasing the proportion of genotyped animals resulted in higher EBV accuracies, which in turn increased genetic gains. Consequently, genotyping all the available animals led to maximal genetic gain. Similarly, the growing number of animals in the reference population resulted in an increase in genetic gain with every additional breeding cycle irrespective of the scenario. Compared with the reference scenario Ped, the greatest marginal genetic gain was achieved when the top 25% of male lambs were genotyped. These findings agreed with those in the literature (Granleese et al., 2019; Horton et al., 2015). In the simulation study of Granleese et al. (2019), overall increases in genetic gain ranged from 6% to 68% within the simulated breeding programmes. This finding is consistent with the present study; however, comparing increases in genetic gains with different numbers of genotyped selection candidates is difficult, as the population structure and phenotypic information available at the stage of selection or preselection differ among studies (Granleese et al., 2019; Lillehammer et al., 2020). The main mechanisms by which GS increases genetic gain are either a reduction in generation intervals or an increase in selection accuracy. In this study, increasing the proportion of genotyped lambs between scenarios increased the accuracy of EBVs (see Table 3) resulting in the observed differences in genetic gain between the scenarios, whereas the generation intervals were the same for all the scenarios and thus did not affect genetic gain in this study.

As expected, the relative increase in genetic gain was greater for the health trait with a lower heritability (0.1), and the overall genetic gain was greater for the higher heritable production trait (0.3). The potential of GS, especially for traits with low heritability, has been confirmed in several studies (Kaseja et al., 2023; Rupp et al., 2016; van der Werf, 2009).

Inbreeding rates were lower for the GS scenarios than for the reference scenario with pedigree‐based BLUP breeding value estimation, which has also been reported in other studies (Granleese et al., 2019; Pook et al., 2021). This is because with pedigree‐based selection, individuals are tendentially more selected within families as families tend to have similar breeding values. With GS, this issue is inferior thus leading to more between family selection and reducing inbreeding. The lower rates of inbreeding with GS due to a better prediction of the Mendelian sampling term component of breeding values are also described in Daetwyler et al. (2007). However, given the slow increase in reference population size and the high genetic diversity in the German Merino breed (Schmid et al., 2023), the number of simulated breeding cycles was obviously too small to detect more pronounced differences between scenarios concerning inbreeding rates.

### 4.2 Transfer of the simulated scenarios to the actual breeding programme

The simulation was designed to mimic the actual breeding programme of the German Merino breed as realistically as possible; thus, a realistic population size, number of individuals per cohort and genome size were assumed. However, some simplifications had to be made to capture the impact of different genotyping strategies in a maximally comprehensive set‐up. The main simplification was that only two traits were simulated, and selection was based on a TMI with the same weight for both traits. This enabled a straightforward comparison and excluded the impact of different weights on the breeding goal, which was intended to clearly highlight the effects of different proportions of genotyped individuals on the breeding outcome. In practice, 19 traits are currently implemented in the German Merino sheep breeding programme, and selection is based on a TMI with economically derived weights for each of the traits. Briefly, the wool, muscle conformation and body conformation traits are phenotyped as own performance of lambs before being selected for herdbook‐registration (female) and licensing (male) in a practical breeding scheme. Traits of reproduction, fattening and slaughtering performance are available later in life either as own performance records of ewes (number of lambs born, nursing ability) or as progeny records of rams (e.g. average daily gain, back muscle area). For more details on the currently practiced performance testing, see Martin et al. (2023). As the influence of genotyping strategies was similar for both traits in the simulation study, it can be expected that the general findings might also hold true when more traits are included in the TMI.

So far, no health trait has been addressed in the current breeding goal of German Merino sheep; however, we chose to simulate this relationship regarding the increasing importance of balancing production improvement and health in sustainable breeding goals. The importance of breeding for health traits in sheep was described by Bishop et al. (2002) with gastrointestinal parasite infestations being the most important health issue. Breeding for gastrointestinal parasite resistance has already been implemented in breeding programmes (e.g. Brown et al., 2006; Dodds et al., 2014) and the genetic parameters of possible indicator traits of parasite resistance as well as performance traits have been estimated for the German sheep population (Gauly et al., 2002; Gauly & Erhardt, 2001; Schmid et al., 2023). The heritabilities of the simulated health and production traits were derived from these studies, and a slightly negative correlation between the traits, as frequently observed in animal breeding (e.g. Koeck et al., 2012; Medrado et al., 2021), was assumed. Consequently, these two traits can be considered representative of the complexes of health and production.

Genomic information in breeding programmes is particularly beneficial for traits for which phenotypes are available late in life or not for all animals owing to the reduction in generation intervals. As both traits were recorded at the same timepoint and own performance was measured, generation intervals did not affect genetic gain in this simulation. However, generation intervals would still have an impact on real breeding programmes when the phenotyping timepoint differs between traits or when different sources of information (i.e. own and offspring performance) are used. Additionally, early‐life traits that are genetically correlated with the target traits may impact EBV estimation in real breeding programmes, as their information can be used. Because this is rather trait specific, such aspects were not modelled in the present study, which was intended to infer the role of genotype information in a more general view.

The individuals to be genotyped were preselected on the basis of a ssGBLUP breeding value estimation but without their own genotypes of the selection candidates. Another possible choice of animals to be genotyped was a random selection of lambs. This was also evaluated and led to a lower increase in genetic gain compared with the investigated preselection scenarios (not shown elsewhere). Nevertheless, preselection is more likely to be implemented in practice because it is considered economically realistic (Lillehammer et al., 2020). Horton et al. (2015) also suggested two‐stage selection for Australian breeding nucleus rams with the initial selection step only on the basis of non‐genomic information available, thus limiting the cost of genotyping to solely the superior rams. Similarly, the willingness to genotype low‐performing animals in the German low‐input sheep breeding system with limited financial possibilities is considered to be unrealistic. In addition, lambs (both female and male) are usually preselected by breeders at the farm level before eventually being licensed (male) or herdbook‐registered (female) in Germany. Nonselected animals are used for the sale of lamb meat and are, therefore, not available for genotyping in many cases.

To initialize a reference population at the starting point of the GS scenarios, all available individuals of the breeding ram cohort (*n* = 469) were genotyped in breeding cycle 0. In every subsequent breeding cycle, the genotyped candidates were added to the reference population after phenotyping, which thus grew continuously and was maximal at the end of the simulation. We assume this to be appropriate in practice because the reference population is expected to grow slowly in actual German Merino breeding programmes. Accumulating all the data and maximizing the number of individuals in the reference population may be a suitable strategy to maximize profit through the use of genotype information, at least in the short‐ and mid‐term.

The results of this study provide an informative basis for discussions on possible ways to implement GS in German Merino sheep breeding programmes with the aim of accelerating genetic gain. Before practical implementation, detailed economic evaluations are required to derive the real benefits of GS by weighing costs to benefits, as genotyping costs need to be counterbalanced by an increase in genetic gain (van der Werf et al., 2014). The relative benefit of genotyping was greatest in the GSTop25 scenario, which is favourable from a practical and economical point of view because only 25% of the male lambs need to be genotyped. Note that only the herdbook population was simulated in this study; thus, the transfer of genetic gain to production herds by selling rams was not addressed. This should be considered in the context of quantifying the economic profit of GS since the majority of Merino individuals are held in production herds and a large part of the economic benefit of GS is realized in these herds. However, to assess the cost‐effectiveness of the investigated scenarios, the genotyping costs and economic values of the selection traits need to be considered. Such considerations were beyond the scope of this study.

Within the German Merino herdbook population (i.e. the breeding nucleus), herds are genetically connected through sires. It is very common that young rams are sold to other breeders before their first use; however, ram‐rotation after use is not practiced. As described in Lillehammer et al. (2020) and valid for German sheep breeding, dams are selected within herds, and selection decisions and mating decisions are made by farmers. This, in combination with the absence of AI use or sire‐rotation, may increase the importance of considering flock structures in breeding programmes. However, the consideration of flock structure in the simulation would have required knowledge about the actual genetic connectedness between flocks for which no valid data were available.

Finally, a certain infrastructure for the genomic breeding programme must be guaranteed prior to implementation (Simianer et al., 2019). This may include routine sampling and genotyping at practical, logistical and institutional levels as well as the connection of genotyping, performance testing and breeding value estimation. As German sheep breeding works in a low‐input system that involves harsh environments and a large workload, straightforward and cost‐efficient processes are essential, starting with the easy handling of the genotype sampling of selection candidates through ear tagging (e.g. Ossowski et al., 2022).

## 5 CONCLUSION

This simulation study evaluated the potential of GS for the German Merino sheep breeding programme by comparing different ssGBLUP strategies to a reference scenario with pedigree‐based BLUP breeding value estimation. It was confirmed that genetic gain increased with increasing number of genotyped lambs and was more beneficial when heritability was low. The results revealed that genotyping all the animals maximizes the genetic gain and accuracy of the breeding values; however, genotyping a 25% proportion of the male lambs preselected on the basis of EBVs was shown to have the greatest relative benefit on these metrics. This study provides a comprehensive basis for discussions on genotyping strategies for ssGBLUP in German Merino sheep breeding.

## FUNDING INFORMATION

This research received no external funding.

## CONFLICT OF INTEREST STATEMENT

The authors declare that there are no conflicts of interest.

## Supporting information


Data S1.

Table S1.

## Data Availability

All simulations were run via MoBPS version 1.11.06. The simulation script and software are available at https://github.com/tpook92/MoBPS and in Data S1. Genetic data are available from the corresponding author upon request.
