# Supplementary material for: Genomic selection strategies for the German Merino sheep breeding programme – A simulation study
Source: J Anim Breed Genet. 2024 Sep 11;142(3):251–62. doi: 10.1111/jbg.12897 (PMC11975160; doi:10.1111/jbg.12897)
Supplement: Supplementary file 2 — Table S1. [file JBG-142-251-s001.docx]

*Supplementary Tables S1 to S12*

**Genomic selection strategies for the German Merino sheep breeding program: A simulation study**

R. Martin ^1^, T. Pook ^2^, J. Bennewitz ^1^, M. Schmid ^1^

^1^ *Institute of Animal Science, University of Hohenheim, Garbenstr. 17, 70599 Stuttgart, Germany*

^2^ *Animal Breeding and Genomics, Wageningen University & Research, P.O. Box 388, 6700AH Wageningen, The Netherlands*

Corresponding author: Rebecca Martin. Email: rebecca.martin@uni-hohenheim.de

**Supplementary Table S1**

Mean true breeding values and standard deviations (SD) across all 100 simulated runs per breeding cycle in the considered breeding cycles 1 to 10 for the health trait for the reference scenario Ped (pedigree-based breeding value estimation) and the alternative scenarios with genomic selection strategies GSTop25, GSTop50, GS100, GS100+Top25, GS100+Top50 and GS100+100 for the breeding ram cohort and contrast significances between scenarios

| **.**  **.** | **Scenario** | | | | | | | | | | | | | |
| --- | --- | --- | --- | --- | --- | --- | --- | --- | --- | --- | --- | --- | --- | --- |
| **.**  **.** | **Ped^a^** | | **GSTop25^b^** | | **GSTop50^c^** | | **GS100^d^** | | **GS100+Top25^e^** | | **GS100+Top50^e^** | | **GS100+100^e^** | |
| **Breeding cycle** | **Mean** | **SD** | **Mean** | **SD** | **Mean** | **SD** | **Mean** | **SD** | **Mean** | **SD** | **Mean** | **SD** | **Mean** | **SD** |
| 1 | 0.131 | 0.040 | 0.110 | 0.038 | 0.094 | 0.044 | 0.118 | 0.041 | 0.118 | 0.042 | 0.120 | 0.040 | 0.132 | 0.040 |
| 2 | 0.272 | 0.062 | 0.255 | 0.064 | 0.244 | 0.063 | 0.294 | 0.063 | 0.302 | 0.062 | 0.303 | 0.062 | 0.319 | 0.060 |
| 3 | 0.412 | 0.073 | 0.408 | 0.076 | 0.421 | 0.077 | 0.476 | 0.074 | 0.491 | 0.072 | 0.493 | 0.070 | 0.514 | 0.072 |
| 4 | 0.554 | 0.084 | 0.576 | 0.082 | 0.595 | 0.090 | 0.661 | 0.088 | 0.683 | 0.086 | 0.682 | 0.076 | 0.702 | 0.084 |
| 5 | 0.697 | 0.095 | 0.739 | 0.094 | 0.766 | 0.107 | 0.846 | 0.103 | 0.875 | 0.097 | 0.877 | 0.085 | 0.893 | 0.097 |
| 6 | 0.833 | 0.106 | 0.896 | 0.108 | 0.941 | 0.127 | 1.025 | 0.115 | 1.065 | 0.113 | 1.072 | 0.100 | 1.084 | 0.108 |
| 7 | 0.967 | 0.125 | 1.061 | 0.121 | 1.111 | 0.135 | 1.205 | 0.122 | 1.255 | 0.125 | 1.260 | 0.107 | 1.281 | 0.122 |
| 8 | 1.103 | 0.139 | 1.220 | 0.129 | 1.283 | 0.147 | 1.377 | 0.129 | 1.447 | 0.129 | 1.446 | 0.112 | 1.472 | 0.135 |
| 9 | 1.241 | 0.151 | 1.385 | 0.136 | 1.460 | 0.157 | 1.555 | 0.140 | 1.638 | 0.139 | 1.643 | 0.123 | 1.658 | 0.148 |
| 10 | 1.380 | 0.160 | 1.554 | 0.141 | 1.632 | 0.163 | 1.734 | 0.149 | 1.832 | 0.142 | 1.839 | 0.131 | 1.852 | 0.155 |

a-e Scenarios with different superscripts differ significantly at *P* < 0.05.

**Supplementary Table S2**

Mean true breeding values and standard deviations (SD) across all 100 simulated runs per breeding cycle in the considered breeding cycles 1 to 10 for the production trait for the reference scenario Ped (pedigree-based breeding value estimation) and the alternative scenarios with genomic selection strategies GSTop25, GSTop50, GS100, GS100+Top25, GS100+Top50 and GS100+100 for the breeding ram cohort and contrast significances between scenarios

| **.**  **.** | **Scenario** | | | | | | | | | | | | | |
| --- | --- | --- | --- | --- | --- | --- | --- | --- | --- | --- | --- | --- | --- | --- |
| **.**  **.** | **Ped^a^** | | **GSTop25^b^** | | **GSTop50^c^** | | **GS100^d^** | | **GS100+Top25^e^** | | **GS100+Top50^e^** | | **GS100+100^e^** | |
| **Breeding cycle** | **Mean** | **SD** | **Mean** | **SD** | **Mean** | **SD** | **Mean** | **SD** | **Mean** | **SD** | **Mean** | **SD** | **Mean** | **SD** |
| 1 | 0.197 | 0.040 | 0.168 | 0.039 | 0.150 | 0.04 | 0.172 | 0.041 | 0.174 | 0.042 | 0.176 | 0.042 | 0.187 | 0.042 |
| 2 | 0.394 | 0.061 | 0.361 | 0.054 | 0.365 | 0.052 | 0.406 | 0.057 | 0.428 | 0.060 | 0.419 | 0.056 | 0.431 | 0.056 |
| 3 | 0.585 | 0.071 | 0.579 | 0.058 | 0.602 | 0.063 | 0.648 | 0.066 | 0.686 | 0.072 | 0.678 | 0.065 | 0.696 | 0.066 |
| 4 | 0.778 | 0.081 | 0.798 | 0.065 | 0.830 | 0.074 | 0.890 | 0.075 | 0.940 | 0.082 | 0.934 | 0.069 | 0.954 | 0.078 |
| 5 | 0.970 | 0.090 | 1.024 | 0.076 | 1.061 | 0.080 | 1.126 | 0.083 | 1.193 | 0.085 | 1.193 | 0.080 | 1.208 | 0.084 |
| 6 | 1.159 | 0.100 | 1.253 | 0.091 | 1.296 | 0.089 | 1.365 | 0.090 | 1.453 | 0.093 | 1.458 | 0.088 | 1.471 | 0.096 |
| 7 | 1.352 | 0.106 | 1.488 | 0.098 | 1.531 | 0.090 | 1.609 | 0.090 | 1.718 | 0.094 | 1.722 | 0.101 | 1.733 | 0.104 |
| 8 | 1.545 | 0.110 | 1.722 | 0.106 | 1.774 | 0.096 | 1.855 | 0.103 | 1.989 | 0.105 | 1.989 | 0.111 | 2.003 | 0.111 |
| 9 | 1.737 | 0.112 | 1.950 | 0.114 | 2.012 | 0.101 | 2.095 | 0.112 | 2.259 | 0.107 | 2.262 | 0.120 | 2.268 | 0.113 |
| 10 | 1.931 | 0.120 | 2.183 | 0.122 | 2.256 | 0.108 | 2.335 | 0.115 | 2.518 | 0.116 | 2.534 | 0.123 | 2.536 | 0.120 |

a-e Scenarios with different superscripts differ significantly at *P* < 0.05.

**Supplementary Table S3**

Mean true breeding values and standard deviations (SD) across all 100 simulated runs per breeding cycle in the considered breeding cycles 1 to 10 for the health trait for the reference scenario Ped (pedigree-based breeding value estimation) and the alternative scenarios with genomic selection strategies GSTop25, GSTop50, GS100, GS100+Top25, GS100+Top50 and GS100+100 for the breeding ewe cohort and contrast significances between scenarios

| **.**  **.** | **Scenario** | | | | | | | | | | | | | |
| --- | --- | --- | --- | --- | --- | --- | --- | --- | --- | --- | --- | --- | --- | --- |
| **.**  **.** | **Ped^a^** | | **GSTop25^b^** | | **GSTop50^c^** | | **GS100^d^** | | **GS100+Top25^e^** | | **GS100+Top50^f^** | | **GS100+100^f^** | |
| **Breeding cycle** | **Mean** | **SD** | **Mean** | **SD** | **Mean** | **SD** | **Mean** | **SD** | **Mean** | **SD** | **Mean** | **SD** | **Mean** | **SD** |
| 1 | 0.140 | 0.014 | 0.137 | 0.014 | 0.138 | 0.014 | 0.137 | 0.013 | 0.139 | 0.014 | 0.137 | 0.013 | 0.129 | 0.014 |
| 2 | 0.283 | 0.027 | 0.278 | 0.025 | 0.278 | 0.024 | 0.285 | 0.025 | 0.291 | 0.026 | 0.293 | 0.026 | 0.288 | 0.025 |
| 3 | 0.424 | 0.038 | 0.419 | 0.034 | 0.420 | 0.036 | 0.436 | 0.035 | 0.450 | 0.037 | 0.453 | 0.035 | 0.453 | 0.035 |
| 4 | 0.564 | 0.050 | 0.561 | 0.043 | 0.565 | 0.046 | 0.590 | 0.045 | 0.613 | 0.046 | 0.620 | 0.046 | 0.623 | 0.044 |
| 5 | 0.704 | 0.060 | 0.707 | 0.054 | 0.714 | 0.058 | 0.749 | 0.056 | 0.782 | 0.056 | 0.793 | 0.054 | 0.798 | 0.055 |
| 6 | 0.843 | 0.070 | 0.856 | 0.062 | 0.867 | 0.066 | 0.911 | 0.067 | 0.956 | 0.065 | 0.970 | 0.061 | 0.977 | 0.064 |
| 7 | 0.982 | 0.079 | 1.005 | 0.070 | 1.021 | 0.077 | 1.073 | 0.076 | 1.131 | 0.075 | 1.150 | 0.070 | 1.160 | 0.072 |
| 8 | 1.120 | 0.089 | 1.156 | 0.079 | 1.177 | 0.087 | 1.239 | 0.086 | 1.308 | 0.084 | 1.334 | 0.078 | 1.344 | 0.078 |
| 9 | 1.259 | 0.099 | 1.309 | 0.088 | 1.337 | 0.096 | 1.406 | 0.096 | 1.490 | 0.091 | 1.519 | 0.084 | 1.531 | 0.086 |
| 10 | 1.397 | 0.108 | 1.463 | 0.096 | 1.502 | 0.108 | 1.576 | 0.105 | 1.674 | 0.101 | 1.707 | 0.089 | 1.721 | 0.096 |

a-f Scenarios with different superscripts differ significantly at *P* < 0.05.

**Supplementary Table S4**

Mean true breeding values and standard deviations (SD) across all 100 simulated runs per breeding cycle in the considered breeding cycles 1 to 10 for the production trait for the reference scenario Ped (pedigree-based breeding value estimation) and the alternative scenarios with genomic selection strategies GSTop25, GSTop50, GS100, GS100+Top25, GS100+Top50, and GS100+100 for the breeding ewe cohort and contrast significances between scenarios

| **.**  **.** | **Scenario** | | | | | | | | | | | | | |
| --- | --- | --- | --- | --- | --- | --- | --- | --- | --- | --- | --- | --- | --- | --- |
| **.**  **.** | **Ped^a^** | | **GSTop25^b^** | | **GSTop50^c^** | | **GS100^d^** | | **GS100+Top25^e^** | | **GS100+Top50^f^** | | **GS100+100^f^** | |
| **Breeding cycle** | **Mean** | **SD** | **Mean** | **SD** | **Mean** | **SD** | **Mean** | **SD** | **Mean** | **SD** | **Mean** | **SD** | **Mean** | **SD** |
| 1 | 0.191 | 0.014 | 0.193 | 0.015 | 0.191 | 0.015 | 0.185 | 0.015 | 0.189 | 0.015 | 0.186 | 0.015 | 0.175 | 0.015 |
| 2 | 0.387 | 0.026 | 0.384 | 0.025 | 0.381 | 0.025 | 0.382 | 0.025 | 0.394 | 0.026 | 0.395 | 0.027 | 0.388 | 0.026 |
| 3 | 0.580 | 0.039 | 0.575 | 0.035 | 0.575 | 0.036 | 0.582 | 0.035 | 0.607 | 0.037 | 0.612 | 0.037 | 0.609 | 0.036 |
| 4 | 0.774 | 0.047 | 0.769 | 0.044 | 0.774 | 0.045 | 0.788 | 0.044 | 0.827 | 0.046 | 0.837 | 0.048 | 0.838 | 0.045 |
| 5 | 0.966 | 0.057 | 0.966 | 0.053 | 0.976 | 0.053 | 0.997 | 0.053 | 1.054 | 0.054 | 1.069 | 0.057 | 1.074 | 0.054 |
| 6 | 1.158 | 0.066 | 1.168 | 0.059 | 1.181 | 0.062 | 1.209 | 0.061 | 1.285 | 0.063 | 1.307 | 0.065 | 1.314 | 0.062 |
| 7 | 1.349 | 0.074 | 1.373 | 0.069 | 1.390 | 0.071 | 1.426 | 0.069 | 1.521 | 0.072 | 1.551 | 0.074 | 1.561 | 0.071 |
| 8 | 1.542 | 0.080 | 1.582 | 0.078 | 1.604 | 0.079 | 1.647 | 0.076 | 1.764 | 0.080 | 1.801 | 0.084 | 1.811 | 0.080 |
| 9 | 1.736 | 0.086 | 1.795 | 0.087 | 1.823 | 0.086 | 1.872 | 0.083 | 2.011 | 0.089 | 2.054 | 0.093 | 2.065 | 0.090 |
| 10 | 1.928 | 0.094 | 2.012 | 0.096 | 2.044 | 0.094 | 2.099 | 0.089 | 2.264 | 0.097 | 2.312 | 0.102 | 2.323 | 0.098 |

a-f Scenarios with different superscripts differ significantly at *P* < 0.05.

**Supplementary Table S5**

Mean accuracies of breeding value estimation and standard deviations (SD) across all 100 simulated runs per breeding cycle in the considered breeding cycles 1 to 10 for the health trait for the reference scenario Ped (pedigree-based breeding value estimation) and the alternative scenarios with genomic selection strategies GSTop25, GSTop50, GS100, GS100+Top25, GS100+Top50 and GS100+100 for the breeding ram cohort and contrast significances between scenarios

| **.**  **.** | **Scenario** | | | | | | | | | | | | | |
| --- | --- | --- | --- | --- | --- | --- | --- | --- | --- | --- | --- | --- | --- | --- |
| **.**  **.** | **Ped^a^** | | **GSTop25^b^** | | **GSTop50^bc^** | | **GS100^c^** | | **GS100+Top25^d^** | | **GS100+Top50^d^** | | **GS100+100^d^** | |
| **Breeding cycle** | **Mean** | **SD** | **Mean** | **SD** | **Mean** | **SD** | **Mean** | **SD** | **Mean** | **SD** | **Mean** | **SD** | **Mean** | **SD** |
| 1 | 0.535 | 0.044 | 0.512 | 0.054 | 0.503 | 0.058 | 0.527 | 0.052 | 0.527 | 0.053 | 0.531 | 0.052 | 0.540 | 0.051 |
| 2 | 0.527 | 0.053 | 0.535 | 0.049 | 0.545 | 0.049 | 0.569 | 0.046 | 0.578 | 0.043 | 0.583 | 0.046 | 0.590 | 0.041 |
| 3 | 0.526 | 0.045 | 0.549 | 0.048 | 0.560 | 0.046 | 0.582 | 0.045 | 0.595 | 0.039 | 0.593 | 0.043 | 0.601 | 0.044 |
| 4 | 0.529 | 0.047 | 0.560 | 0.050 | 0.573 | 0.046 | 0.587 | 0.051 | 0.602 | 0.040 | 0.607 | 0.037 | 0.607 | 0.042 |
| 5 | 0.525 | 0.052 | 0.567 | 0.046 | 0.579 | 0.044 | 0.591 | 0.043 | 0.609 | 0.041 | 0.615 | 0.038 | 0.616 | 0.040 |
| 6 | 0.520 | 0.053 | 0.567 | 0.045 | 0.585 | 0.041 | 0.592 | 0.044 | 0.614 | 0.041 | 0.616 | 0.043 | 0.620 | 0.036 |
| 7 | 0.516 | 0.046 | 0.568 | 0.045 | 0.587 | 0.042 | 0.596 | 0.040 | 0.614 | 0.037 | 0.618 | 0.034 | 0.623 | 0.036 |
| 8 | 0.526 | 0.041 | 0.571 | 0.047 | 0.589 | 0.043 | 0.599 | 0.041 | 0.620 | 0.035 | 0.617 | 0.035 | 0.621 | 0.041 |
| 9 | 0.524 | 0.050 | 0.579 | 0.042 | 0.590 | 0.043 | 0.602 | 0.042 | 0.623 | 0.034 | 0.621 | 0.038 | 0.623 | 0.044 |
| 10 | 0.526 | 0.047 | 0.585 | 0.046 | 0.596 | 0.045 | 0.601 | 0.043 | 0.621 | 0.037 | 0.624 | 0.036 | 0.623 | 0.042 |

a-d Scenarios with different superscripts differ significantly at *P* < 0.05.

**Supplementary Table S6**

Mean accuracies of breeding value estimation and standard deviations (SD) across all 100 simulated runs per breeding cycle in the considered breeding cycles 1 to 10 for the production trait for the reference scenario Ped (pedigree-based breeding value estimation) and the alternative scenarios with genomic selection strategies GSTop25, GSTop50, GS100, GS100+Top25, GS100+Top50 and GS100+100 for the breeding ram cohort and contrast significances between scenarios

| **.**  **.** | **Scenario** | | | | | | | | | | | | | |
| --- | --- | --- | --- | --- | --- | --- | --- | --- | --- | --- | --- | --- | --- | --- |
| **.**  **.** | **Ped^a^** | | **GSTop25^b^** | | **GSTop50^c^** | | **GS100^c^** | | **GS100+Top25^d^** | | **GS100+Top50^d^** | | **GS100+100^d^** | |
| **Breeding cycle** | **Mean** | **SD** | **Mean** | **SD** | **Mean** | **SD** | **Mean** | **SD** | **Mean** | **SD** | **Mean** | **SD** | **Mean** | **SD** |
| 1 | 0.687 | 0.032 | 0.676 | 0.032 | 0.662 | 0.033 | 0.685 | 0.032 | 0.687 | 0.032 | 0.693 | 0.031 | 0.703 | 0.029 |
| 2 | 0.685 | 0.032 | 0.693 | 0.030 | 0.707 | 0.029 | 0.726 | 0.028 | 0.741 | 0.030 | 0.740 | 0.028 | 0.742 | 0.028 |
| 3 | 0.684 | 0.033 | 0.711 | 0.024 | 0.723 | 0.025 | 0.738 | 0.031 | 0.749 | 0.027 | 0.755 | 0.026 | 0.758 | 0.027 |
| 4 | 0.689 | 0.031 | 0.719 | 0.026 | 0.734 | 0.028 | 0.746 | 0.027 | 0.764 | 0.020 | 0.765 | 0.026 | 0.766 | 0.026 |
| 5 | 0.688 | 0.029 | 0.724 | 0.026 | 0.737 | 0.031 | 0.751 | 0.027 | 0.767 | 0.021 | 0.772 | 0.023 | 0.771 | 0.024 |
| 6 | 0.686 | 0.029 | 0.730 | 0.028 | 0.742 | 0.027 | 0.753 | 0.024 | 0.771 | 0.023 | 0.779 | 0.025 | 0.775 | 0.023 |
| 7 | 0.684 | 0.031 | 0.728 | 0.027 | 0.744 | 0.026 | 0.753 | 0.025 | 0.776 | 0.026 | 0.780 | 0.025 | 0.777 | 0.023 |
| 8 | 0.684 | 0.033 | 0.731 | 0.031 | 0.750 | 0.026 | 0.756 | 0.022 | 0.779 | 0.026 | 0.782 | 0.021 | 0.783 | 0.024 |
| 9 | 0.686 | 0.036 | 0.738 | 0.028 | 0.752 | 0.026 | 0.755 | 0.025 | 0.784 | 0.024 | 0.784 | 0.022 | 0.785 | 0.022 |
| 10 | 0.685 | 0.032 | 0.738 | 0.024 | 0.752 | 0.027 | 0.759 | 0.027 | 0.784 | 0.023 | 0.784 | 0.022 | 0.787 | 0.023 |

a-d Scenarios with different superscripts differ significantly at *P* < 0.05.

**Supplementary Table S7**

Mean accuracies of breeding value estimation and standard deviations (SD) across all 100 simulated runs per breeding cycle in the considered breeding cycles 1 to 10 for the health trait for the reference scenario Ped (pedigree-based breeding value estimation) and the alternative scenarios with genomic selection strategies GSTop25, GSTop50, GS100, GS100+Top25, GS100+Top50 and GS100+100 for the breeding ewe cohort and contrast significances between scenarios

| **.**  **.** | **Scenario** | | | | | | | | | | | | | |
| --- | --- | --- | --- | --- | --- | --- | --- | --- | --- | --- | --- | --- | --- | --- |
| **.**  **.** | **Ped^a^** | | **GSTop25^b^** | | **GSTop50^c^** | | **GS100^d^** | | **GS100+Top25^e^** | | **GS100+Top50^ef^** | | **GS100+100^f^** | |
| **Breeding cycle** | **Mean** | **SD** | **Mean** | **SD** | **Mean** | **SD** | **Mean** | **SD** | **Mean** | **SD** | **Mean** | **SD** | **Mean** | **SD** |
| 1 | 0.561 | 0.026 | 0.558 | 0.025 | 0.557 | 0.024 | 0.553 | 0.024 | 0.558 | 0.025 | 0.555 | 0.025 | 0.547 | 0.026 |
| 2 | 0.561 | 0.026 | 0.560 | 0.022 | 0.562 | 0.023 | 0.571 | 0.024 | 0.588 | 0.023 | 0.591 | 0.023 | 0.595 | 0.022 |
| 3 | 0.561 | 0.024 | 0.563 | 0.020 | 0.566 | 0.022 | 0.580 | 0.022 | 0.606 | 0.020 | 0.609 | 0.019 | 0.615 | 0.021 |
| 4 | 0.560 | 0.023 | 0.566 | 0.021 | 0.572 | 0.023 | 0.589 | 0.023 | 0.621 | 0.018 | 0.626 | 0.019 | 0.632 | 0.021 |
| 5 | 0.560 | 0.022 | 0.571 | 0.022 | 0.579 | 0.024 | 0.595 | 0.022 | 0.636 | 0.019 | 0.640 | 0.018 | 0.645 | 0.019 |
| 6 | 0.559 | 0.023 | 0.574 | 0.022 | 0.586 | 0.024 | 0.601 | 0.023 | 0.648 | 0.019 | 0.652 | 0.018 | 0.656 | 0.019 |
| 7 | 0.558 | 0.023 | 0.579 | 0.022 | 0.593 | 0.025 | 0.608 | 0.024 | 0.658 | 0.019 | 0.662 | 0.018 | 0.667 | 0.018 |
| 8 | 0.560 | 0.024 | 0.582 | 0.022 | 0.599 | 0.025 | 0.614 | 0.022 | 0.656 | 0.019 | 0.670 | 0.019 | 0.675 | 0.018 |
| 9 | 0.560 | 0.025 | 0.588 | 0.022 | 0.604 | 0.025 | 0.619 | 0.023 | 0.673 | 0.019 | 0.677 | 0.017 | 0.681 | 0.018 |
| 10 | 0.560 | 0.025 | 0.590 | 0.022 | 0.610 | 0.024 | 0.623 | 0.022 | 0.679 | 0.017 | 0.682 | 0.017 | 0.685 | 0.017 |

a-f Scenarios with different superscripts differ significantly at *P* < 0.05

**Supplementary Table S8**

Mean accuracies of breeding value estimation and standard deviations (SD) across all 100 simulated runs per breeding cycle in the considered breeding cycles 1 to 10 for the production trait for the reference scenario Ped (pedigree-based breeding value estimation) and the alternative scenarios with genomic selection strategies GSTop25, GSTop50, GS100, GS100+Top25, GS100+Top50 and GS100+100 for the breeding ewe cohort and contrast significances between scenarios

| **.**  **.** | **Scenario** | | | | | | | | | | | | | |
| --- | --- | --- | --- | --- | --- | --- | --- | --- | --- | --- | --- | --- | --- | --- |
| **.**  **.** | **Ped^a^** | | **GSTop25^b^** | | **GSTop50^c^** | | **GS100^d^** | | **GS100+Top25^e^** | | **GS100+Top50^f^** | | **GS100+100^f^** | |
| **Breeding cycle** | **Mean** | **SD** | **Mean** | **SD** | **Mean** | **SD** | **Mean** | **SD** | **Mean** | **SD** | **Mean** | **SD** | **Mean** | **SD** |
| 1 | 0.709 | 0.013 | 0.708 | 0.013 | 0.707 | 0.013 | 0.704 | 0.013 | 0.711 | 0.014 | 0.710 | 0.014 | 0.704 | 0.015 |
| 2 | 0.712 | 0.013 | 0.711 | 0.013 | 0.712 | 0.013 | 0.718 | 0.012 | 0.738 | 0.012 | 0.740 | 0.012 | 0.743 | 0.012 |
| 3 | 0.713 | 0.014 | 0.713 | 0.014 | 0.716 | 0.012 | 0.723 | 0.013 | 0.754 | 0.011 | 0.758 | 0.011 | 0.762 | 0.011 |
| 4 | 0.713 | 0.014 | 0.716 | 0.012 | 0.721 | 0.012 | 0.730 | 0.013 | 0.769 | 0.011 | 0.773 | 0.011 | 0.779 | 0.010 |
| 5 | 0.713 | 0.014 | 0.718 | 0.012 | 0.725 | 0.012 | 0.736 | 0.013 | 0.782 | 0.010 | 0.786 | 0.010 | 0.791 | 0.010 |
| 6 | 0.713 | 0.013 | 0.722 | 0.012 | 0.731 | 0.012 | 0.743 | 0.012 | 0.794 | 0.009 | 0.797 | 0.010 | 0.802 | 0.009 |
| 7 | 0.713 | 0.013 | 0.725 | 0.012 | 0.736 | 0.011 | 0.748 | 0.012 | 0.803 | 0.009 | 0.806 | 0.010 | 0.810 | 0.009 |
| 8 | 0.713 | 0.013 | 0.729 | 0.012 | 0.741 | 0.011 | 0.753 | 0.011 | 0.811 | 0.008 | 0.814 | 0.009 | 0.818 | 0.009 |
| 9 | 0.713 | 0.013 | 0.733 | 0.012 | 0.746 | 0.011 | 0.757 | 0.010 | 0.817 | 0.008 | 0.821 | 0.008 | 0.823 | 0.009 |
| 10 | 0.713 | 0.012 | 0.738 | 0.012 | 0.752 | 0.010 | 0.760 | 0.010 | 0.823 | 0.007 | 0.826 | 0.008 | 0.827 | 0.009 |

a-f Scenarios with different superscripts differ significantly at *P* < 0.05.

**Supplementary Table S9**

Mean accuracies of breeding value estimation and standard deviations (SD) across all 100 simulated runs per breeding cycle in the considered breeding cycles 1 to 10 for the health trait for the reference scenario Ped (pedigree-based breeding value estimation) and the alternative scenarios with genomic selection strategies GSTop25, GSTop50, GS100, GS100+Top25, GS100+Top50 and GS100+100 for the male lambs cohort and contrast significances between scenarios

| **.**  **.** | **Scenario** | | | | | | | | | | | | | |
| --- | --- | --- | --- | --- | --- | --- | --- | --- | --- | --- | --- | --- | --- | --- |
| **.**  **.** | **Ped^a^** | | **GSTop25^b^** | | **GSTop50^c^** | | **GS100^d^** | | **GS100+Top25^e^** | | **GS100+Top50^e^** | | **GS100+100^e^** | |
| **Breeding cycle** | **Mean** | **SD** | **Mean** | **SD** | **Mean** | **SD** | **Mean** | **SD** | **Mean** | **SD** | **Mean** | **SD** | **Mean** | **SD** |
| 1 | 0.417 | 0.024 | 0.423 | 0.026 | 0.434 | 0.027 | 0.464 | 0.026 | 0.474 | 0.026 | 0.479 | 0.026 | 0.487 | 0.024 |
| 2 | 0.416 | 0.024 | 0.429 | 0.024 | 0.446 | 0.025 | 0.482 | 0.027 | 0.497 | 0.021 | 0.500 | 0.023 | 0.505 | 0.023 |
| 3 | 0.414 | 0.025 | 0.434 | 0.024 | 0.456 | 0.024 | 0.494 | 0.027 | 0.513 | 0.021 | 0.515 | 0.022 | 0.523 | 0.023 |
| 4 | 0.413 | 0.025 | 0.441 | 0.027 | 0.462 | 0.024 | 0.504 | 0.025 | 0.526 | 0.021 | 0.530 | 0.021 | 0.533 | 0.023 |
| 5 | 0.415 | 0.028 | 0.443 | 0.026 | 0.469 | 0.024 | 0.510 | 0.027 | 0.539 | 0.021 | 0.540 | 0.020 | 0.543 | 0.019 |
| 6 | 0.414 | 0.026 | 0.446 | 0.025 | 0.477 | 0.026 | 0.518 | 0.023 | 0.547 | 0.019 | 0.549 | 0.021 | 0.553 | 0.018 |
| 7 | 0.411 | 0.024 | 0.448 | 0.025 | 0.482 | 0.023 | 0.524 | 0.023 | 0.552 | 0.020 | 0.555 | 0.020 | 0.559 | 0.019 |
| 8 | 0.413 | 0.026 | 0.456 | 0.024 | 0.486 | 0.024 | 0.530 | 0.022 | 0.560 | 0.018 | 0.562 | 0.019 | 0.565 | 0.020 |
| 9 | 0.412 | 0.025 | 0.458 | 0.023 | 0.493 | 0.024 | 0.536 | 0.024 | 0.566 | 0.017 | 0.569 | 0.020 | 0.570 | 0.020 |
| 10 | 0.410 | 0.026 | 0.459 | 0.025 | 0.495 | 0.024 | 0.540 | 0.022 | 0.572 | 0.020 | 0.571 | 0.020 | 0.575 | 0.021 |

a-e Scenarios with different superscripts differ significantly at *P* < 0.05

**Supplementary Table S10**

Mean accuracies of breeding value estimation and standard deviations (SD) across all 100 simulated runs per breeding cycle in the considered breeding cycles 1 to 10 for the production trait for the reference scenario Ped (pedigree-based breeding value estimation) and the alternative scenarios with genomic selection strategies GSTop25, GSTop50, GS100, GS100+Top25, GS100+Top50 and GS100+100 for the male lambs cohort and contrast significances between scenarios

| **.**  **.** | **Scenario** | | | | | | | | | | | | | |
| --- | --- | --- | --- | --- | --- | --- | --- | --- | --- | --- | --- | --- | --- | --- |
| **.**  **.** | **Ped^a^** | | **GSTop25^b^** | | **GSTop50^c^** | | **GS100^d^** | | **GS100+Top25^e^** | | **GS100+Top50^ef^** | | **GS100+100^f^** | |
| **Breeding cycle** | **Mean** | **SD** | **Mean** | **SD** | **Mean** | **SD** | **Mean** | **SD** | **Mean** | **SD** | **Mean** | **SD** | **Mean** | **SD** |
| 1 | 0.534 | 0.018 | 0.541 | 0.019 | 0.557 | 0.019 | 0.592 | 0.017 | 0.610 | 0.018 | 0.612 | 0.015 | 0.620 | 0.017 |
| 2 | 0.533 | 0.018 | 0.549 | 0.018 | 0.570 | 0.017 | 0.613 | 0.016 | 0.634 | 0.017 | 0.638 | 0.016 | 0.645 | 0.016 |
| 3 | 0.534 | 0.018 | 0.553 | 0.018 | 0.581 | 0.017 | 0.627 | 0.017 | 0.656 | 0.016 | 0.658 | 0.014 | 0.664 | 0.014 |
| 4 | 0.535 | 0.018 | 0.560 | 0.018 | 0.590 | 0.017 | 0.638 | 0.016 | 0.670 | 0.013 | 0.673 | 0.013 | 0.676 | 0.014 |
| 5 | 0.535 | 0.017 | 0.564 | 0.018 | 0.598 | 0.017 | 0.651 | 0.013 | 0.683 | 0.013 | 0.685 | 0.013 | 0.689 | 0.014 |
| 6 | 0.533 | 0.017 | 0.569 | 0.018 | 0.605 | 0.016 | 0.657 | 0.015 | 0.692 | 0.013 | 0.695 | 0.014 | 0.698 | 0.013 |
| 7 | 0.533 | 0.017 | 0.572 | 0.018 | 0.610 | 0.016 | 0.663 | 0.014 | 0.702 | 0.012 | 0.704 | 0.013 | 0.707 | 0.013 |
| 8 | 0.535 | 0.018 | 0.577 | 0.016 | 0.618 | 0.015 | 0.669 | 0.013 | 0.709 | 0.013 | 0.711 | 0.012 | 0.714 | 0.013 |
| 9 | 0.533 | 0.020 | 0.581 | 0.016 | 0.623 | 0.017 | 0.675 | 0.013 | 0.715 | 0.013 | 0.717 | 0.013 | 0.719 | 0.011 |
| 10 | 0.534 | 0.020 | 0.586 | 0.016 | 0.627 | 0.016 | 0.682 | 0.013 | 0.721 | 0.012 | 0.722 | 0.012 | 0.725 | 0.012 |

a-f Scenarios with different superscripts differ significantly at *P* < 0.05.

**Supplementary Table S11**

Mean accuracies of breeding value estimation and standard deviations (SD) across all 100 simulated runs per breeding cycle in the considered breeding cycles 1 to 10 for the health trait for the reference scenario Ped (pedigree-based breeding value estimation) and the alternative scenarios with genomic selection strategies GSTop25, GSTop50, GS100, GS100+Top25, GS100+Top50 and GS100+100 for the female lambs cohort and contrast significances between scenarios

| **.**  **.** | **Scenario** | | | | | | | | | | | | | |
| --- | --- | --- | --- | --- | --- | --- | --- | --- | --- | --- | --- | --- | --- | --- |
| **.**  **.** | **Ped^a^** | | **GSTop25^b^** | | **GSTop50^c^** | | **GS100^d^** | | **GS100+Top25^e^** | | **GS100+Top50^f^** | | **GS100+100^g^** | |
| **Breeding cycle** | **Mean** | **SD** | **Mean** | **SD** | **Mean** | **SD** | **Mean** | **SD** | **Mean** | **SD** | **Mean** | **SD** | **Mean** | **SD** |
| 1 | 0.417 | 0.025 | 0.417 | 0.024 | 0.421 | 0.028 | 0.432 | 0.026 | 0.447 | 0.026 | 0.465 | 0.026 | 0.486 | 0.026 |
| 2 | 0.415 | 0.025 | 0.420 | 0.026 | 0.425 | 0.026 | 0.439 | 0.026 | 0.461 | 0.023 | 0.477 | 0.023 | 0.505 | 0.024 |
| 3 | 0.414 | 0.025 | .0423 | 0.026 | 0.432 | 0.023 | 0.444 | 0.025 | 0.471 | 0.021 | 0.490 | 0.023 | 0.520 | 0.023 |
| 4 | 0.413 | 0.024 | 0.428 | 0.027 | 0.434 | 0.024 | 0.446 | 0.025 | 0.481 | 0.022 | 0.502 | 0.023 | 0.532 | 0.023 |
| 5 | 0.413 | 0.027 | 0.429 | 0.025 | 0.438 | 0.026 | 0.448 | 0.025 | 0.489 | 0.022 | 0.510 | 0.021 | 0.544 | 0.020 |
| 6 | 0.411 | 0.025 | 0.432 | 0.026 | 0.441 | 0.028 | 0.452 | 0.023 | 0.496 | 0.023 | 0.518 | 0.021 | 0.553 | 0.020 |
| 7 | 0.412 | 0.025 | 0.433 | 0.025 | 0.446 | 0.026 | 0.455 | 0.021 | 0.498 | 0.022 | 0.524 | 0.021 | 0.560 | 0.019 |
| 8 | 0.414 | 0.026 | 0.439 | 0.023 | 0.450 | 0.025 | 0.458 | 0.023 | 0.504 | 0.021 | 0.529 | 0.019 | 0.565 | 0.021 |
| 9 | 0.412 | 0.027 | 0.439 | 0.025 | 0.451 | 0.028 | 0.461 | 0.023 | 0.509 | 0.020 | 0.535 | 0.019 | 0.571 | 0.021 |
| 10 | 0.413 | 0.024 | 0.445 | 0.027 | 0.456 | 0.026 | 0.464 | 0.023 | 0.513 | 0.019 | 0.538 | 0.020 | 0.574 | 0.019 |

a-g Scenarios with different superscripts differ significantly at *P* < 0.05

**Supplementary Table S12**

Mean accuracies of breeding value estimation and standard deviations (SD) across all 100 simulated runs per breeding cycle in the considered breeding cycles 1 to 10 for the production trait for the reference scenario Ped (pedigree-based breeding value estimation) and the alternative scenarios with genomic selection strategies GSTop25, GSTop50, GS100, GS100+Top25, GS100+Top50 and GS100+100 for the female lambs cohort and contrast significances between scenarios

| **.**  **.** | **Scenario** | | | | | | | | | | | | | |
| --- | --- | --- | --- | --- | --- | --- | --- | --- | --- | --- | --- | --- | --- | --- |
| **.**  **.** | **Ped^a^** | | **GSTop25^b^** | | **GSTop50^c^** | | **GS100^d^** | | **GS100+Top25^e^** | | **GS100+Top50^f^** | | **GS100+100^g^** | |
| **Breeding cycle** | **Mean** | **SD** | **Mean** | **SD** | **Mean** | **SD** | **Mean** | **SD** | **Mean** | **SD** | **Mean** | **SD** | **Mean** | **SD** |
| 1 | 0.534 | 0.018 | 0.535 | 0.0019 | 0.537 | 0.019 | 0.545 | 0.018 | 0.571 | 0.018 | 0.590 | 0.016 | 0.620 | 0.017 |
| 2 | 0.532 | 0.019 | 0.537 | 0.018 | 0.544 | 0.016 | 0.549 | 0.019 | 0.585 | 0.018 | 0.608 | 0.016 | 0.645 | 0.015 |
| 3 | 0.535 | 0.019 | 0.542 | 0.017 | 0.547 | 0.017 | 0.554 | 0.019 | 0.594 | 0.017 | 0.621 | 0.015 | 0.664 | 0.015 |
| 4 | 0.534 | 0.019 | 0.543 | 0.018 | 0.552 | 0.018 | 0.559 | 0.017 | 0.606 | 0.014 | 0.633 | 0.015 | 0.676 | 0.013 |
| 5 | 0.533 | 0.017 | 0.548 | 0.019 | 0.556 | 0.020 | 0.563 | 0.016 | 0.613 | 0.014 | 0.644 | 0.016 | 0.689 | 0.012 |
| 6 | 0.533 | 0.018 | 0.549 | 0.019 | 0.559 | 0.017 | 0.568 | 0.017 | 0.621 | 0.014 | 0.653 | 0.015 | 0.698 | 0.013 |
| 7 | 0.534 | 0.018 | 0.551 | 0.018 | 0.561 | 0.018 | 0.570 | 0.018 | 0.628 | 0.012 | 0.660 | 0.015 | 0.707 | 0.014 |
| 8 | 0.533 | 0.018 | 0.554 | 0.016 | 0.565 | 0.016 | 0.572 | 0.018 | 0.635 | 0.013 | 0.666 | 0.013 | 0.713 | 0.013 |
| 9 | 0.535 | 0.019 | 0.561 | 0.017 | 0.570 | 0.016 | 0.576 | 0.015 | 0.638 | 0.014 | 0.671 | 0.012 | 0.718 | 0.012 |
| 10 | 0.533 | 0.020 | 0.561 | 0.017 | 0.573 | 0.018 | 0.578 | 0.016 | 0.642 | 0.013 | 0.675 | 0.012 | 0.724 | 0.013 |

a-g Scenarios with different superscripts differ significantly at *P* < 0.05.
